# Independent Manipulation of Topological Charges and Polarization Patterns of Optical Vortices

**DOI:** 10.1038/srep31546

**Published:** 2016-08-16

**Authors:** Ching-Han Yang, Yuan-Di Chen, Shing-Trong Wu, Andy Ying-Guey Fuh

**Affiliations:** 1 Department of Photonics, National Cheng Kung University, Tainan 701, Taiwan; 2 Laser and Additive Manufacturing Technology Center, Industrial Technology Research Institute, Tainan City 734, Taiwan; 3 Department of Physics, National Cheng Kung University, Tainan 701, Taiwan; 4 Advanced Optoelectronic Technology Center, National Cheng Kung University, Tainan 701, Taiwan

## Abstract

We present a simple and flexible method to generate various vectorial vortex beams (VVBs) with a Pancharatnam phase based on the scheme of double reflections from a single liquid crystal spatial light modulator (SLM). In this configuration, VVBs are constructed by the superposition of two orthogonally polarized orbital angular momentum (OAM) eigenstates. To verify the optical properties of the generated beams, Stokes polarimetry is developed to measure the states of polarization (SOP) over the transverse plane, while a Shack–Hartmann wavefront sensor is used to measure the OAM charge of beams. It is shown that both the simulated and the experimental results are in good qualitative agreement. In addition, polarization patterns and OAM charges of generated beams can be controlled independently using the proposed method.

It is well known that scalar optical vortices having a spatially distributed skew phase front exp(i
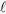
*φ*) have an orbital angular momentum (OAM) of 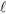
*ħ* per photon, where 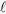 is topological charge^1^. Light-carrying OAM has some practical applications, such as optical communication^2^ and remote sensing^3^. Additionally, if the states of polarization (SOP) of light are space-variant, vectorial vortex beams (VVBs) with polarization singularities appear. A polarization singularity occurs around a point where a scalar vortex is centered in one of the scalar component of VVBs^4^. One interesting property of VVBs is that they can have OAM if a Pancharatnam phase is embedded on the beam^5^. The Pancharatnam phase, also called the Pancharatnam–Berry geometrical phase, usually occurs in the polarization manipulation of ligh^6^,^7^. Some optical devices such as q-plates^8^ or subwavelength elements^9^ based on the Pancharatnam–Berry phase can generate VVBs with the Pancharatnam phase, leading to the carrying of OAM. Recently, the potential for use of VVBs in optical communication has been demonstrated experimentally^10^,^11^.

In principle, VVBs are constructed by superimposing two orthogonally polarized OAM eigenstates with different topological charges. Nowadays, various methods are employed to generate VVBs by using a liquid crystal spatial light modulator (SLM)^12^,^13^,^14^. However, most previous works on this topic discuss cases where the magnitudes of the two topological charges and weights of the two OAM eigenstates are equal^14^,^15^. Although experimental results pertaining to unequal topological charge magnitudes are available, the OAM of light has not been investigated adequately^16^. In this paper, we apply a similar technique as that proposed by Moreno *et al*.^15^,^17^ to study cases where both the topological charges and the weights of two OAM eigenstates are unequal. It is shown that using these degrees of freedom, independent control of polarization patterns and OAM charges of VVBs is possible. In addition, the generated VVBs with a Pancharatnam phase correspond to a coordinate on the hybrid-order Poincaré sphere^18^, which is proposed to describe the evolution of polarization states in inhomogeneous anisotropc media. In the experiment, the panel of a reflective SLM is divided into equal areas, and each of them displays a helical phase hologram to encode OAM eigenstates onto the x- and y-polarized components of incident light, respectively. Next, a quarter-wave plate (QWP) is used to transform the two linear polarization states into another pair of orthogonal polarization states; this step yields two orthogonally polarized OAM eigenstates that further span the VVB subspace. The weighting factors of the two eigenstates can be controlled by adjusting the polarization angle of the incident linearly polarized beam, while the topological charge of each eigenstate can be controlled independently by two separate halves of a single SLM. To evaluate the properties of the generated VVBs, we successively apply two measurement procedures. First, we use Stokes polarimetry^8^,^19^ to study the polarization patterns of the generated beams. Second, we use a Shack–Hartmann wavefront sensor to demonstrate the existence of OAM and further infer the actual OAM charge of beams^20^. Theoretical and experimental results pertaining to SOP and OAM charges are in good agreement.

## Results

### Theoretical description of vectorial vortex beams

The normalized Jones vector of a VVB propagating along the +z direction can be expressed as follows^18^,^21^


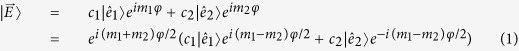


where 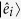 (i = 1, 2) denotes one of orthogonally polarized OAM eigenstates with a topological charge of *m*_*i*_, *φ* is the azimuthal coordinate of the beam, and the weighting factors of each eigenstate are governed by two complex constants *c*_*i*_ = |*c*_*i*_|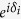. Field normalization requires that


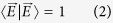



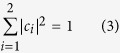


**Figure 1:**
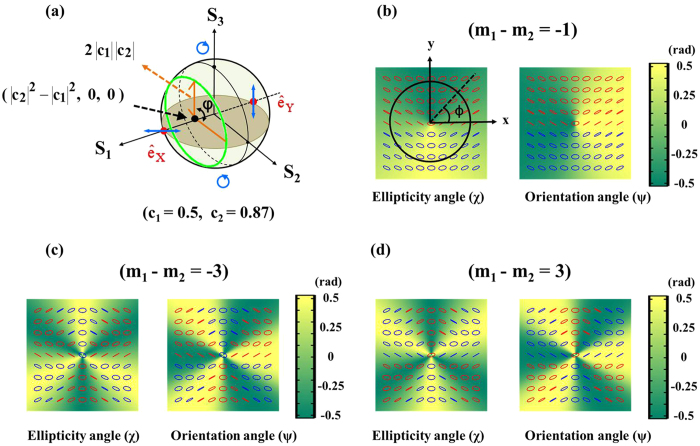
Illustration of using equation (1) to construct VVBs. (**a**) In this example, polarization eigenstates of 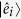 in equation (1) are chosen as linearly polarized states, denoted by 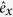 and 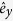, respectively. The two weighting coefficients are *c*_1_ = 0.5 and *c*_2_ = 0.87, respectively. The geodesic path (green line) marked on the Poincaré sphere describes the SOP on the transverse plane. (**b–d**) Simulation results of ellipticity and orientation angles of polarization ellipses. The blue and red ellipses on the color maps represent right- and left-handed polarizations, respectively.

For the benefit of the reader, we briefly outline the approach to constructing VVBs by using equation (1). For simplicity, we first consider a special case where 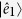 = 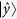 and 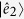 = 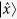, that is, two orthogonal linear polarization eigenstates. By substituting equation (1) into the definition of Stokes parameters, we obtain


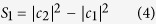



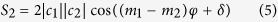



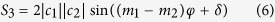


where*δ* ≡ *δ*_1_ − *δ*_2_ is the phase difference between the two complex constants of c_i_, and it is related to the choice of origin of the azimuthal *φ*-coordinate. Equations (4)–(6), 14, 15 imply that all SOP on the transverse plane can be described completely by a geodesic path with a radius of 2|*c*_1_||*c*_2_|, located on the plane of intersection of *S*_1_ = |*c*_2_|^2^ − |*c*_1_|^2^ with the Poincaré sphere, as shown in Fig. 1(a). Moreover, both *S*_2_ and *S*_3_ depend only on the value of (*m*_1_ − *m*_2_) and, therefore, SOP does, too. The fact of SOP depend only on (*m*_1_ − *m*_2_) can also be found in equation (1) because the sum of *m*_1_ and *m*_2_ is only relevant to the common phase factor of the two orthogonal eigenstates. To control SOP, one can adjust the coefficients of *c*_*i*_, which would result in shifting of the geodesic path on the sphere, or adjust the value of (*m*_1_ − *m*_2_). Figure 1(b–d) show the simulated results of the orientation angle (*ψ*) and the ellipticity angle (*χ*) of SOP for *c*_1_ = 0.5 and *c*_2_ = 0.87, but different values of (*m*_1_ − *m*_2_) = 1, −3, and 3. These results were obtained by substituting equations (4)–(6), 14, 15 into equations (23) and (24), where the angle *ψ* (−*π*/2 ≤ *ψ* ≤ *π*/2) determines the direction of the major axis of the polarization ellipses, and *χ* (−*π*/4 ≤ *χ* ≤ *π*/4) determines the ratio of the minor to the major axes of the ellipses. In addition, *χ* is positive for right-handed polarizations (blue ellipses) but negative for left-handed (red ellipses). By comparing Fig. 1(b–d), it can be seen that the azimuthal gradient of SOP depends on the absolute value of (*m*_1_ − *m*_2_) while the distribution of handedness depends on its sign. Furthermore, the distribution of SOP depends only on the azimuthal angle of *φ* because there is only one spatial variable of *φ* in equation (1). Although we discuss only orthogonal linear polarization eigenstates, similar discussions can be applied to another pair of orthogonally polarized eigenstates. For detailed discussions, please refer to the supplementary information.

The average OAM charge 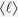 in equation (1) can be found by examining the ratio of the z component of OAM to its energy over the transverse plane of light^22^. This is given as follows


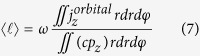



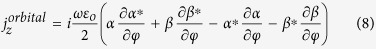



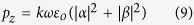


where c is the speed of light in free space, *ω* is the angular frequency of light, *j*_*z*_ is the z component of OAM density, *p*_*z*_ is the z component of linear momentum density, and *α* and *β* are complex amplitudes of the x and y electric field components of the VVBs^22^. In fact, it is shown in the supplementary information that the average OAM charge in equation (1) is independent of the selection of polarization eigenstates of 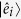. As proved in the supplementary information, the average OAM charge is


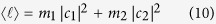


One can also find the Pancharatnam phase *φ*_*p*_ in equation (1) from the following definition^5^,^22^,^23^


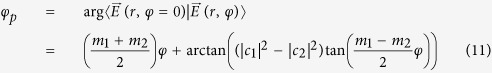


where 
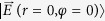
 is the reference field, and the operator “arg” denotes the argument of the inner product. By comparing equations (10) and (11), it can be found that if *c*_1_ = *c*_2_ and *m*_1_ = −*m*_2_, either the Pancharatnam phase or the OAM charge vanishes, while other opposite cases are true. From above discussions, we conclude that SOP depend on the value of (*m*_1_ − *m*_2_), as well as on the two selected orthogonal polarization eigenstates, whereas OAM charge depends not only on the weighting coefficients but also on the values of *m*_1_ and *m*_2_. Consequently, independent control of OAM charges and polarization patterns of VVBs can be achieved by manipulating various parameters. Details of the relationship between the OAM charge and the Pancharatnam phase can be found in the literature^22^.

### Experimental setup

**Figure 2:**
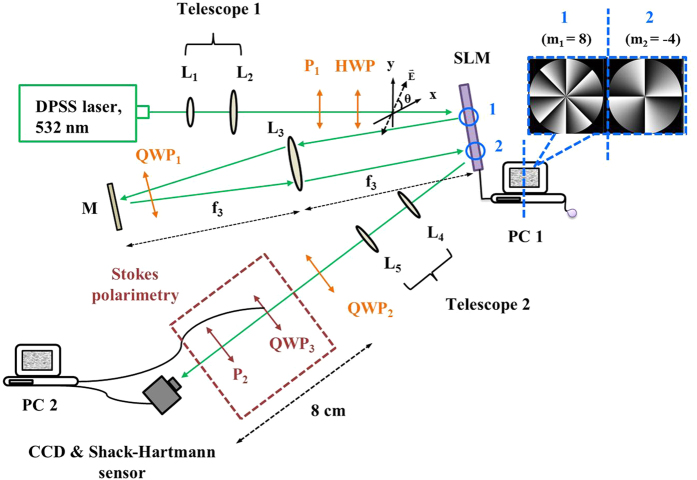
Experimental setup of the double modulation scheme. L: lens, P: polarizer, HWP: half-wave late, QWP: quarter-wave plate, M: mirror. The inset at right-top corner shows an example of displaying two helical phase holograms with different topological charges of (*m*_1_, *m*_2_) = (8, −4) onto the SLM.

The double modulation scheme is shown in Fig. 2. A diode-pumped solid-state (DPSS) laser beam (Verdi, *λ* = 532 nm) is filtered and expanded by the first telescope consisting of lens *L*_1_ (*f*_1_ = 15 mm) and *L*_2_ (*f*_2_ = 150 mm) and then passes through a polarizer (*P*_1_) and an *HWP*. *P*_1_ is introduced to generate a linearly polarized beam, and the *HWP* is used to adjust the polarization plane of the linearly polarized beam. The beam with its polarization making an angle *θ* with the x-axis is then incident on the half panel of a phase-only reflective SLM (Holoeye Photomics, PLUTO-VIS, 1920 × 1080 pixels) with LC molecules aligned in the x direction. The extinction ratio of the SLM is about −18 dB. Two different helical phase holograms with 900 × 900 pixels are simultaneously displayed side by side onto the SLM to independently encode the x and y electric field components of the incident beam. After the first reflection is generated on area 1, where only the x component is modulated, the beam is then imaged onto area 2 through a reflective type 4f (*f*_3_ = 75 cm) system in which *QWP*_1_ is inserted to spatially rotate the polarization state by 90°. After reflection from area 2, where, again, only the x component is modulated, the beam is then imaged onto *QWP*_2_ by using a second telescope consisting of *L*_4_ (*f*_4_ = 20 cm) and *L*_5_ (*f*_5_ = 30 cm). *QWP*_2_ may be rotated to transform two linearly polarized OAM eigenstates into another pair of orthogonally polarized OAM eigenstates and attain the VVBs. Finally, the generated beams are passed through Stokes polarimetry consisting of *QWP*_3_ and polarizer *P*_2_, the transmission axis of which is fixed on the x-axis. *QWP*_3_ is electrically controlled by a computer, and the resolution is set to be 3°. A CCD camera (Newport LBP-4-USB) is used to record the variation of intensity distributions as *QWP*_3_ is rotates by 360°. Hence, the point-to-point Stokes parameters on the beams cross section can be obtained. Afterwards, Stokes polarimetry is removed and the CCD camera is replaced by a Shack-Hartmann wavefront sensor (Throlabs, WFS150-5C camera mounted with AR coated lens arrays at a pitch 300 μm and effective focal length of 14 mm) to measure the OAM charge of VVBs.

### Theoretical modeling of vectorial vortex beam generation

To begin with, we briefly review the fundamental principle of the double modulation scheme^17^. Because of the phase-only modulation effect of reflective SLM, the LC molecules are aligned in a parallel fashion in the x-direction relative to the laboratory reference frame. As a result, only the x component of the reflected modulated beam carries the designed phase information, while the y component does not. To encode the y component, the polarization state must be rotated by 90° so that the x and y components are inverted. This explains why the modulation scheme uses both a SLM that is divided into two equal areas to modulate the incident beam twice and a reflective-type 4f system combined with a QWP to rotate the polarization state, as shown in Fig. 2. It should be mentioned that the extinction ratio of our calibrated SLM is about −18 dB (shown in the supplementary information) and, therefore, the y component will also be phase modulated in each modulation process. This may cause small errors in our experimental results.

As demonstrated in the supplementary information, the Jones vector of the resultant modulated-beam after passing through the double modulation system is given by


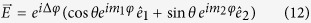


where 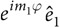 and 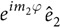 are two orthogonally polarized OAM eigenstates with topological charges of *m*_1_ and *m*_2_, respectively. Δ*φ* denotes the relative phase offset between the eigenstates; thus, it is related to the choice of origin of the azimuthal *φ*-coordinate. As described in Fig. 2, *θ* is the angle between the polarization of the initial linearly polarized beam and the x-axis, and it controls the weights of the two orthogonal eigenstates. A comparison of equations (1) and (12) reveals that *c*_1_ = cos *θ* and *c*_2_ = sin *θ*, respectively; therefore, a theoretical OAM charge can be obtained by substituting *c*_1_ and *c*_2_ into equation (10). The polarization patterns of the VVBs generated based on equation (12) are measured experimentally by Stokes polarimetry. The point-to-point Stokes parameters over the transverse plane can be retrieved from several intensity patterns recorded using a CCD camera (details are given in the methods section). For measuring the OAM charge, Stokes polarimetry is replaced with a Shack–Hartmann sensor. The sensor consists of an array of lenses of the same focal length such that each microlens generates a spot on the sensor. The spot shift between the actual spot position and its corresponding reference position is proportional to the local skew angle of the Poynting vectors with respect to a beam axis^20^,^24^,^25^. Thus, this non-interferometric technique enables us to infer an actual OAM charge of VVBs.

### Generation results and discussion

As mentioned previously, the *QWP*_2_ in Fig. 2 is employed to control the polarizations of two orthogonal OAM eigenstates, and the *HWP* controls their weighting factors. The experimental results of three different polarization eigenstates are presented in the following subsections.

#### Circular polarization eigenstates

**Figure 3:**
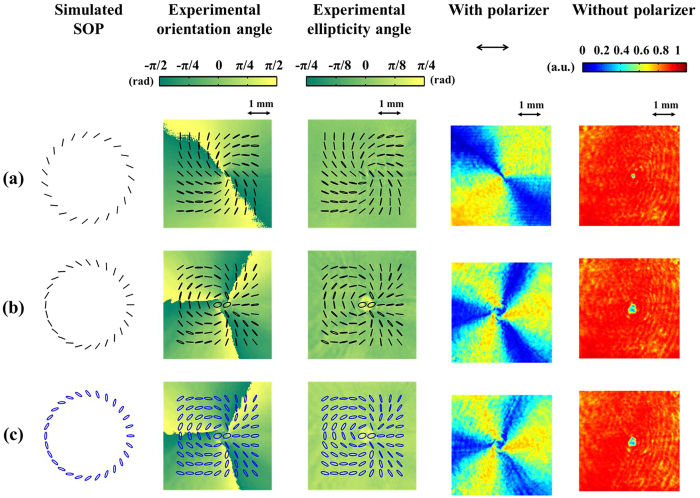
VVBs constructed by circularly polarized OAM eigenstates. The first column shows simulated SOP, where the black and blue marks represent linear and right-handed polarization states, respectively. The second and third columns show measured SOP with angles of orientation and ellipticity of polarization ellipses. The fourth column shows the transmitted intensity patterns of light behind a polarizer with its transmission axis in the x direction. The last column shows intensity patterns of light without passing through a polarizer. Associated parameters in each row are (**a**) (*m*_1_, *m*_2_) = (−1, 1), *θ* = 45°, and Δ*φ* = 90°, (**b**) (*m*_1_, *m*_2_) = (3, 6), *θ* = 45°, and Δ*φ* = 0°, (**c**) (*m*_1_, *m*_2_) = (3, 6), *θ* = 30°, and Δ*φ* = 0°.

In this subsection, the slow axis of *QWP*_2_ in Fig. 2 is set to 45° such that 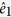 and 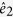 in equation (12) are the right-handed circular polarization (RCP) and left-handed circular polarization (LCP) states, respectively. Figure 3 shows the corresponding experimental results obtained using different parameters. Here, black and blue marks in the first, second, and third columns represent the ideally linear and right-handed polarization states, respectively. The first row of the figure shows a generally polarized CVB. The second and third rows show the results obtained using higher indexes of *m*_1_ and *m*_2_. A comparison of simulated and experimentally measured SOP reveals that they are in good qualitative agreement for all results. At the same time, we also show the measured results of orientation and ellipticity angles of polarization ellipses in the second and third columns. The fourth column shows the intensity patterns of light behind a polarizer. The patterns obtained exhibit three extinction regions on the beam cross section. Our simulations indicate that, as a general rule, the total number of angularly distributed extinction regions around the beam axis is |*m*_1_ − *m*_2_|. Moreover, when the value of *θ* deviates from 45°, the contrast of the intensity pattern in the fourth column reduces because of the impurity of the linear polarization states. The last column shows the intensity patterns of light without passing through a polarizer; all of the intensity patterns obtained display a donut-shaped distribution.

#### Linear polarization eigenstates

**Figure 4:**
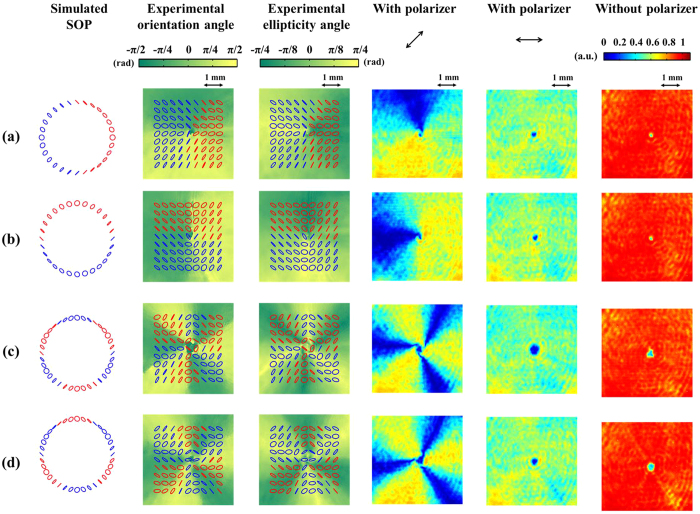
VVBs constructed by linearly polarized OAM eigenstates. The first column shows simulated SOP, where the blue and red ellipses, respectively, represent the right- and left-handed polarization states. The second and third columns show measured SOP with angles of orientation and ellipticity of polarization ellipses. The fourth and fifth columns show transmitted intensity patterns of light behind a polarizer with its transmission axis in the 45° and 0° directions, respectively. The last column shows intensity patterns of light without passing through a polarizer. Associated parameters in each row are (**a**) (*m*_1_, *m*_2_) = (1, 2), *θ* = 45°, and Δ*φ* = −90°, (**b**) (*m*_1_, *m*_2_) = (1, 2), *θ* = 45°, and Δ*φ* = 0°, (**c**) (*m*_1_, *m*_2_) = (3, 6), *θ* = 45°, and Δ*φ* = 0°, (**d**) (*m*_1_, *m*_2_) = (6, 3), *θ* = 45°, and Δ*φ* = 0°.

In this subsection, the slow axis of *QWP*_2_ in Fig. 2 is set to 0° such that 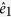 and 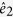 are the y- and x- linearly polarized eigenstates, respectively. To understand how SOP is affected by Δ*φ*, Fig. 4(a,b) show the experimental results obtained using (*m*_1_, *m*_2_) = (1, 2), *θ* = 45°, and different Δ*φ* values. Figure 4(c,d) show the experimental results obtained with higher indexes of (*m*_1_, *m*_2_). In addition, we consider the effect of swapping the indexes of (*m*_1_, *m*_2_) on SOP. A comparison of Fig. 4(a,b) reveals that a change in Δ*φ* influences the spatial rotation of SOP with respect to a beam axis. We carried out a series of simulations (results not shown) and found that the polarization distribution on the beam rotates through an angle Δ*φ*/(|*m*_1_ − |*m*_2_|) about a beam axis for a given shifted value of Δ*φ*; this distribution shows counterclockwise and clockwise rotations, respectively, for positive and negative values of Δ*φ*. A comparison of Fig. 4(c,d) reveals that swapping the indexes of (*m*_1_, *m*_2_) leads to inversion of the handedness of SOP, but the intensity patterns remain the same regardless of the presence of a polarizer. A comparison of the simulated and experimentally obtained SOP reveals that they are in good qualitative agreement. The fourth and fifth columns show the intensity patterns of light behind a polarizer with its transmission axis at 45° and 0°, respectively. As can be seen, when the polarizer axis is set to 45°, which is orthogonal to some local linear polarization states on the beam, the contrast of the intensity patterns is high. However, as indicated in the fifth column, this contrast disappears when the polarizer is oriented to 0° because the polarizer is no longer orthogonal to any local polarization state. The last column shows the intensity patterns of light that has not passed through a polarizer. As in the case of circular eigenstates, all obtained patterns feature a donut-shaped intensity distribution.

#### Elliptical polarization eigenstates

**Figure 5:**
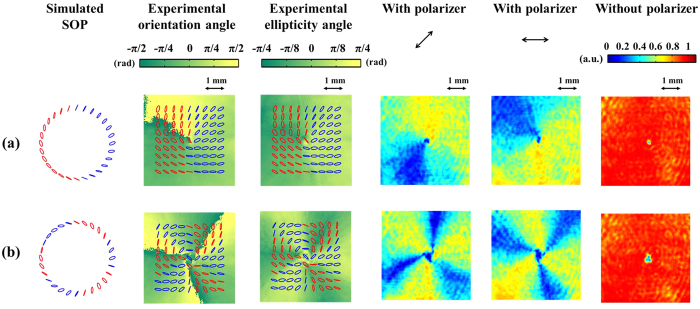
VVBs constructed by elliptically polarized OAM eigenstates. The first column shows the simulated SOP, where the blue and red ellipses are the right- and left- handed polarization states, respectively. The second and third columns show measured SOP with angles of orientation and ellipticity of polarization ellipses. The fourth and fifth columns show transmitted intensity patterns of light behind a polarizer with its transmission axis in the 45° and 0° directions, respectively. The last column shows intensity patterns of light without passing through a polarizer. Associated parameters in each row are (**a**) (m_1_, m_2_) = (1, 2), *θ* = 45°, and Δ*φ* = 0°, (**b**) (m_1_, m_2_) = (3, 6), *θ* = 45°, and Δ*φ* = 0°.

In this subsection, the slow axis of *QWP*_2_ in Fig. 2 is set to 22.5°, and the corresponding eigenstates are sketched in the supplementary Fig. S5 online. The effect of introducing Δ*φ* is similar (results not shown) to those of the linear eigenstates. The experimental results of different indexes of (*m*_1_, *m*_2_) where *θ* = 45° and Δ*φ* = 0° are shown in Fig. 5. There is a good agreement between the simulated and experimentally measured SOP in the figure. The fourth and fifth columns show the intensity patterns of light behind a polarizer with its transmission axis respectively set to 45° and 0° with respect to the x-axis. The intensity patterns depend on the orientation of a polarizer, as in the case of linear eigenstates. The last column shows the intensity patterns of light that has not passed through a polarizer. Similar to previous results, there is a donut-shaped intensity distribution when a polarizer is not used.

Thus far, we have considered only the SOP of VVBs. Another important characteristic of light is the OAM charge. As pointed out in equation (11), the Pancharatnam phase can be divided into two independent parts, and each of which is discussed in the following subsections.

• Case with *θ* = 45°.

In the case of *θ* = 45°, equally weighted eigenstates leading the Pancharatnam phase and OAM charge are as follows

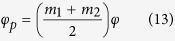

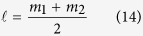


**Figure 6:**
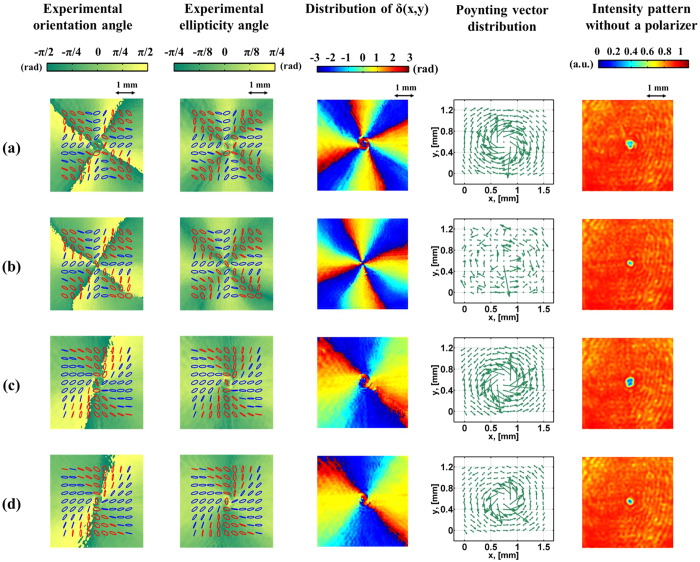
SOP and Poynting vector distributions of VVBs at elliptical eigenstates for *θ* = 45°. The first two columns show the measured SOP, as well as the orientation and ellipticity angles of polarization ellipses. The blue and red ellipses are the right- and left-handed polarization states, respectively. The third column shows the distribution of *δ* (x, y). The fourth column shows the displacements of focal spots on the Shack–Hartmann sensor plane. The last column shows the intensity patterns of light without passing through a polarizer. Associated parameters in each row are (**a**) (*m*_1_, *m*_2_) = (−3, −7), 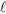 = −5, (**b**) (*m*_1_, *m*_2_) = (2, −2), 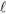 = 0, (**c**) (*m*_1_, *m*_2_) = (4, 6), 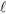 = 5, (**d**) (*m*_1_, *m*_2_) = (2, 4), 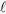 = 3. All of the above parameters correspond to the same values of *θ* = 45° and Δ*φ* = 0°.

**Figure 7:**
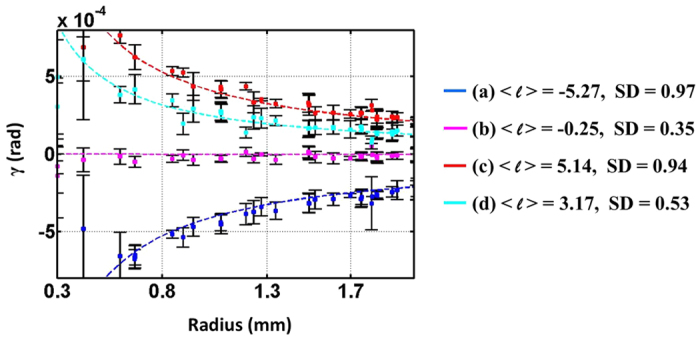
Measured skew angle *γ* of Poynting vectors at different beam radii. Each curve corresponds to each row of Fig. 6. The average OAM charge and standard deviation are listed on the side.

The experimental results of different sets of (*m*_1_, *m*_2_) are shown in Fig. 6. Figure 6(a,b) correspond to the same differences but different sums of (*m*_1_, *m*_2_); in particular, the sum of *m*_1_ and *m*_2_ in Fig. 6(b) is zero. Figure 6(c,d) correspond to another pair of values of sums and differences. The first two columns of Fig. 6 show the measured SOP, as well as the orientation and ellipticity angles of the polarization ellipses. Those figures with the same values of (*m*_1_ − *m*_2_) reveal the same SOP, as indicated by equation (1). In practice, referring to Fig. 2, the distance from *QWP*_3_, in which the VVBs are generated, through Stokes polarimetry to the CCD camera is about 8 cm; hence, SOP evolution during propagation cannot be ignored^13^. This condition is confirmed by the measured phase difference *δ* (x, y) on the transverse plane shown in the third column, which is obtained by substituting the measured Stokes parameters into supplementary equations (S4) and (S5). The structure of *δ* (x, y) around the beam center is distorted for 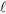 ≠ 0, but it remains stable otherwise. The fourth column shows the displacement of focal spots between the actual spot position and its corresponding reference position, as measured by the Shack–Hartmann sensor. A substantial amount of curl around the beam axis for 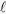 ≠ 0 may be observed; the opposite is true when 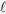 is zero. The length and direction of each arrow correspond to the projection of the local Poynting vector onto the wavefront sensor plane, which is perpendicular to the beam axis^20^. Hence, for higher values of 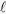, the azimuthal component of the local Poynting vector is larger, yielding longer displacement of the spot shifts pointing toward the azimuthal direction. A comparison of Fig. 6(a,c) reveals that the opposite sign of 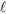 yields the reverse spatial evolution of the Poynting vector. The last column shows the intensity patterns obtained by using a conventional CCD camera instead of Stokes polarimetry. These patterns show that the size of the central dark spot increases with increasing sum of the modulus of *m*_1_ and *m*_2_, as will be discussed in the next subsection. The screw angle (*γ* ≡ 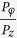 = 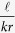), which is the angle between the *φ*- and z- components of the Poynting vectors^20^, can be obtained by projecting the transverse displacement of the focused spots onto the *φ* direction with respect to the beam axis, and subsequently dividing it by the focal length of the lens arrays of the wavefront sensor. A comparison between the theoretical and the measured screw angles at different beam radii is shown in Fig. 7, where the dashed lines (square points) are theoretical (measured) values of *γ*, and each color corresponds to each row of Fig. 6. As can be seen, there is good agreement between the theoretical and the measured results at all positions other than those close to the central singularity of a beam. The average OAM charge obtained by averaging the measurements for radii of 0.2 mm to 2 mm together with the corresponding standard deviation (SD) are listed on the side of Fig. 7. It can be found that the value of SD is proportional to 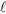 due to the larger central dark spot at the beam center yielding to inaccurate measurement.

• Case with *m*_1_ = −*m*_2_.

In the case of m_1_ = −m_2_, the Pancharatnam phase and OAM charge are


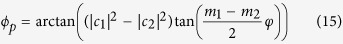



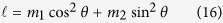


**Figure 8:**
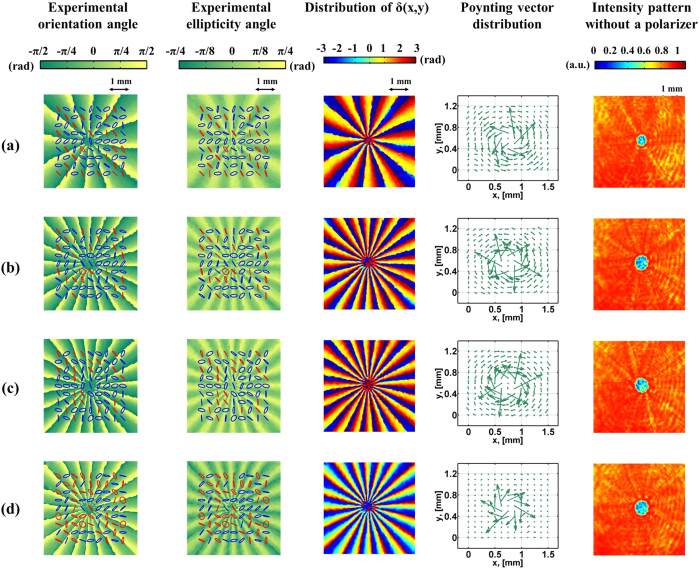
SOP and Poynting vector distributions of VVBs at elliptical eigenstates for *m*_1_ = −*m*_2_. The first two columns show the measured SOP, as well as the orientation and ellipticity angles of polarization ellipses. The blue and red ellipses are the right- and left-handed polarization states, respectively. The third column shows the distribution of *δ* (x, y). The fourth column shows the displacements of focal spots on the Shack–Hartmann sensor plane. The last column shows the intensity patterns of light without passing through a polarizer. Associated parameters in each row are (**a**) (m_1_, m_2_) = (6, −6), *θ* = 30°, and 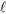 = 3, (**b**) (m_1_, m_2_) = (10, −10), *θ* = 30°, and 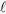 = 5, (**c**) (m_1_, m_2_) = (−10, 10), *θ* = 30°, and 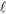 = −5, (**d**) (m_1_, m_2_) = (10, −10), *θ* = 45°, and 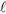 = 0. All of the above parameters correspond to the same value of Δ*φ* = 0°.

**Figure 9:**
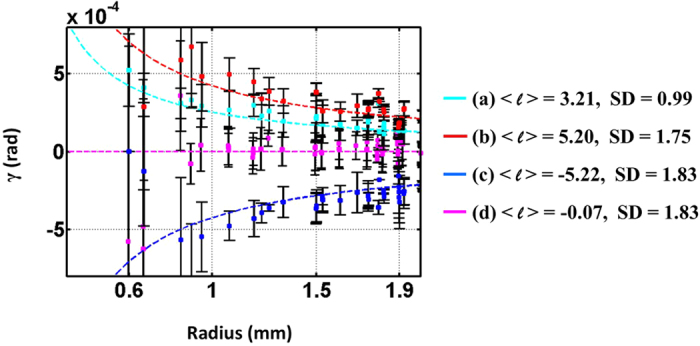
Measured skew angle *γ* of Poynting vectors at different beam radii. Each curve corresponds to each row of Fig. 8. The average OAM charge and standard deviation are listed on the side.

Both of them now depend on the weighted coefficients as well as the values of (*m*_1_, *m*_2_). The experimental results of different sets of (*m*_1_, *m*_2_) are shown in Fig 8. Figure 8(a,b) correspond to completely different values of (*m*_1_ − *m*_2_). Figure 8(b,c) correspond to equal but opposite signs of (*m*_1_ − *m*_2_). By contrast, the value of *θ* in Fig. 8(d) is specifically set to 45°, and the theoretical value of 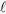 is zero. The first two columns of Fig. 8 show the measured SOP. The second column shows the distribution of *δ* (x, y) on the transverse plane. No distortion of *δ* (x, y) around the beam center is observed, regardless of the value of 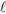; by contrast, as seen in the third column of Fig. 6, only when 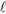 is zero *δ* (x, y) is not distorted. Actually, this distortion is due to the different Gouy phases of the two OAM eigenstates^26^,^27^. The fourth column of Fig. 8 shows the displacement of focal spots as measured by the Shack–Hartmann sensor. As expected, higher values of 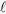 result in larger amounts of curl around a beam axis. The opposite sign of 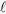 also leads to the reverse spatial evolution of the Poynting vectors. The last column presents the intensity patterns of light without passing through a polarizer. Similar to the previous fifth columns of Fig. 6, the size of the central dark spot increases as the sum of the modulus of *m*_1_ and *m*_2_ increases, likely because of the diffraction behavior of each OAM eigenstate. Based on the diffraction theory^28^, the larger the topological charge of the OAM mode, the larger is the local spatial frequency and the more likely it is that rays with larger skew angles with respect to the z-axis will be produced during propagation. Thus, larger values of |*m*_1_ + *m*_2_| yield larger radii of the central dark spot. This inference can be verified by comparing the fourth columns of Figs 6(b) and 8(d). These two cases correspond to the zero value of 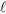 and show no obvious azimuthal component of the Poynting vectors. However, the energy spread of light in the radial direction is significant in the latter case because of the larger value of |*m*_1_ + *m*_2_|. Figure 9shows the measured screw angles of Poynting vectors at different beam radii, where different colors correspond to the respective rows of Fig. 8. As can be seen, there is good agreement between the theoretical (dashed lines) and the measured results (square points). The fluctuation for each curve is larger when the measurements are close to the central point, where the intensity is too low yielding to inaccurate measurement. Finally, it should be mentioned that while only VVBs constructed by two orthogonally polarized elliptical OAM eigenstates are described in this work, similar results can also be obtained for another pair of orthogonal polarization states.

## Conclusion

In this paper, we successfully generated a variety of VVBs with different polarization patterns and OAM charges based on the double reflection of a single SLM. The polarization patterns of the generated fields were analyzed by using Stokes polarimetry, while OAM charge was measured using a Shack–Hartmann wavefront sensor. We confirmed that the SOP around the beam axis becomes unstable during wave propagation if the Gouy phases of the two OAM eigenstates are unequal. Both the experimentally measured SOP and OAM charge of the generated VVBs are close to the theoretical values. In addition, we demonstrated that both polarization patterns and OAM charges can be controlled individually by using the proposed system.

## Methods

### Measurement of the Stokes parameters

The passage of light through an optical element may change its polarization state. Using Stokes parameters, the action of optical elements on the Stokes parameters can be completely described in the Stokes space. In this representation, a polarization state is represented by a Stokes vector, as expressed in equation (17), and the matrix of the optical element is represented by the Mueller matrix. Therefore, the Stokes vector 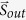 of the outgoing beam can be obtained by carrying out matrix multiplication, as given in equation (18).


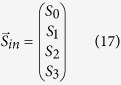



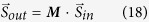


where S_i_ (i = 0, …, 3) in equation (17) are the Stokes parameters of the incident light. As shown in Fig. 2, VBBs to be analyzed is sent through a rotating *QWP*_3_ and then through a polarizer *P*_2_ whose transmission axis is fixed along the x-axis. Subsequently, a CCD camera is used to record the beam intensity as a function of the rotation angle of *QWP*_3_. Thus, the Stokes vector of the outgoing beam passing through Stokes polarimetry can be obtained by


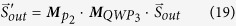


where 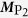 and 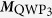 in equation (19) can be written as,


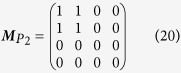



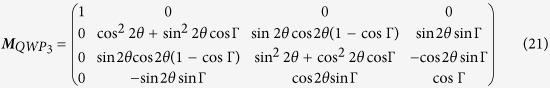


where 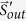 and 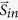 represent the Stokes vectors of the incident and outgoing beams, respectively, 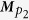 is the Muller matrix of the polarizer *P*_2_, 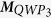 is the Mueller matrix of the *QWP*_3_, the phase retardation Γ of which is *π*/2, and the slow axis of which makes an angle *θ* with the x-axis. The intensity pattern of *I*(*θ*) recorded on the camera is closely related to the first element of 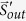. After some algebra, the intensity variation versus rotation angle of *θ* presents the following form


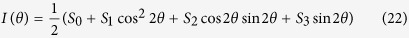


Therefore, the Stokes parameters of VVBs can be obtained by using Fourier series analysis of the intensity pattern I(*θ*). We emphasize here that, because VVBs possess different SOP on the transverse plane, point-to-point Fourier series analysis must be performed over the entire x–y plane. When the Stokes parameters are obtained, other polarization parameters, such as the orientation and ellipticity angles of polarization ellipses, *ψ* and *χ*, can also be obtained by


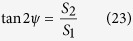



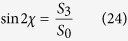


## Additional Information

**How to cite this article**: Yang, C.-H. *et al*. Independent Manipulation of Topological Charges and Polarization Patterns of Optical Vortices. *Sci. Rep.* **6**, 31546; doi: 10.1038/srep31546 (2016).

## Supplementary Material

Supplementary Information
