# Supplementary material for: Independent Manipulation of Topological Charges and Polarization Patterns of Optical Vortices
Source: Sci Rep. 2016 Aug 16;6:31546. doi: 10.1038/srep31546 (PMC4985747; doi:10.1038/srep31546)
Supplement: Supplementary Information [file srep31546-s1.pdf]

Supplementary Information

Independent Manipulation of Topological Charges and Polarization Patterns of Optical Vortices

Ching-Han Yang[1], Yuan-Di Chen[2], Shing-Trong Wu[3], and Andy Ying-Guey Fuh[1,3,4,*]

[1]*Department of Photonics, National Cheng Kung University, Tainan 701, Taiwan*
[2]*Laser and Additive Manufacturing Technology Center, Industrial Technology Research Institute, Tainan City 734, Taiwan*
[3]*Department of Physics, National Cheng Kung University, Tainan 701, Taiwan*
[4]*Advanced Optoelectronic Technology Center, National Cheng Kung University, Tainan 701, Taiwan*
**Corresponding email: andyfuh@mail.ncku.edu.tw*

A. Review of the Jones vector, Stokes parameters, Stokes vector, and Poincaré sphere

The Jones vector is very convenient for describing the polarization state of a polarized beam [S1]. In this representation, the complex Jones vector of a homogeneous polarized beam traveling along the +z direction is written as

$$\vec{J} = \begin{bmatrix} A_x\, e^{i\delta_x} \\ A_y\, e^{i\delta_y} \end{bmatrix} \tag{S1}$$

where A_x and A_y are the amplitudes of the x and y components, respectively, and δ_x and δ_y are their corresponding phases. The polarization state of light can be completely described by two independent parameters: the amplitude ratio, which is defined by $R \equiv A_y/A_x$, and the phase difference between them, which is defined by $\delta \equiv \delta_y - \delta_x$. Stokes showed that the state of a polarized beam can be expressed in terms of four parameters as defined by the following equations

$$S_0 \equiv A_x^2 + A_y^2 \tag{S2}$$

$$S_1 \equiv A_x^2 - A_y^2 \tag{S3}$$

$$S_2 \equiv 2\, A_x A_y \cos\delta \tag{S4}$$

$$S_3 \equiv 2\, A_x A_y \sin\delta \tag{S5}$$

In this representation, S_0 describes the total intensity of light, S_1 describes the intensity difference between the x- and y-linear polarizations, S_2 describes the intensity difference between ±45° linear polarizations, and S_3 describes the difference between right- and left-handed polarizations. If we use Stokes parameters as the sphere's Cartesian coordinates, a Poincaré sphere can be constructed as shown in Fig. S1. Each point on this sphere represents a unique polarization state. For example, right-handed circular polarization (RCP) is represented by the north pole, and left-handed circular polarization (LCP) is represented by the south pole. The linear polarizations are represented by points in the equatorial plane, and the elliptical states are denoted by the points between the poles and the equatorial plane. Right-handed (left-handed) polarizations always lie above (below) the equatorial plane, and orthogonally polarized states are always located opposite each other in the sphere. The polarization ellipse, illustrated in the inset of Fig. 1, is characterized by two angles: the angle ψ determines the direction of the major axis, whereas the angle χ determines the ellipticity, namely the ratio of the minor to major axis of the ellipse b/a. Both of them can be obtained from Stokes parameters, and the results are given by equations (S6) and (S7). It should be noted that the angle ψ varies between $-\pi/2$ to

$\pi/2$ while χ between $-\pi/4$ to $\pi/4$. Moreover, χ is positive (negative) for right- (left-) handed polarization states.

$$\tan 2\psi = \frac{S_2}{S_1} \tag{S6}$$

$$\sin 2\chi = \frac{S_3}{S_0} \tag{S7}$$

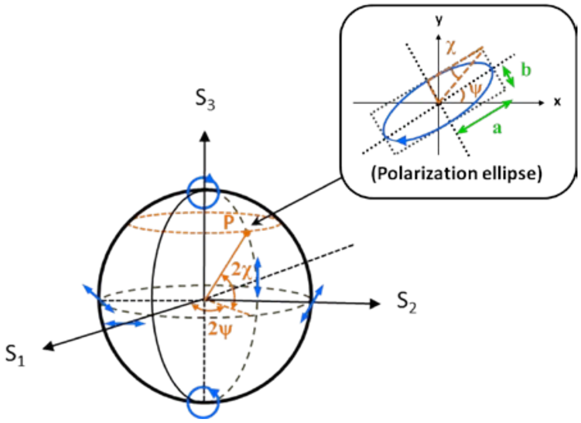

Figure S1. Schematic illustration of a Poincaré sphere. Any elliptical polarization state characterized by its orientation angle (denoted as ψ) and ellipticity angle (denoted as χ) can be geometrically represented as a point on the sphere.

B. Double-modulation scheme for generating vectorial vortex beams

The experimental setup for generating vectorial vortex beams (VVBs) is shown in the main text of Fig. 2. In the setup, we first employ a polarizer (P_1) and a half-wave plate (HWP) pair to produce a linearly polarized beam with its polarization angle oriented at θ with respect to the x axis. Thus, its Jones vector can be written as,

$$\vec{E}_{in} = \begin{bmatrix} E_x \\ E_y \end{bmatrix} = \begin{bmatrix} \cos\theta \\ \sin\theta \end{bmatrix} \tag{S8}$$

During the first modulation, the x component of the electric field is encoded by a phase factor of $e^{i\,p_1(x,y)}$ but the y component is not. Therefore, the Jones vector of the reflected beam is given by

$$\vec{E}_1 = \begin{bmatrix} E_{1,x} \\ E_{1,y} \end{bmatrix} = \begin{bmatrix} -1 & 0 \\ 0 & 1 \end{bmatrix} \cdot \begin{bmatrix} \cos\theta \cdot e^{i\,p_1(x,y)} \\ \sin\theta \end{bmatrix}$$

$$= \begin{bmatrix} -\cos\theta \cdot e^{i\,p_1(x,y)} \\ \sin\theta \end{bmatrix} \tag{S9}$$

where the subscript "1" means the first modulation and $\begin{bmatrix} -1 & 0 \\ 0 & 1 \end{bmatrix}$ represents the reflection off the spatial light modulator (SLM) surface. To encode the y component further, a reflective 4f system consisting of a quarter-wave plate (QWP_1) and a reflective mirror are arranged in a particular order, as shown in Fig. S2. In this arrangement, the Jones matrix is similar to a HWP with its slow axis oriented at 45° except the minus sign in the second row and first column, as derived from equation (S10). In fact, the minus sign indicates that the reflected beam travels in the reverse direction, which means the polarization state can still be rotated by 90°. When calculating equation (S10), careful consideration of the orientation of the slow axis must be made. As shown in Fig. S2, because of mirror reflection, the incident beam sees the slow axis of QWP_1 at 45° while the reflected beam sees the slow axis at 135°.

$$M_{rot} = M_{QWP,135^\circ} \; M_{mirror} \; M_{QWP,45^\circ}$$

$$= \frac{1}{2}\begin{bmatrix} 1 & i \\ i & 1 \end{bmatrix} \cdot \begin{bmatrix} -1 & 0 \\ 0 & 1 \end{bmatrix} \cdot \begin{bmatrix} 1 & -i \\ -i & 1 \end{bmatrix} = \begin{bmatrix} 0 & i \\ -i & 0 \end{bmatrix} \tag{S10}$$

where the subscript "rot" indicates the polarization rotation operation, $M_{QWP,135^\circ}$ and $M_{QWP,45^\circ}$ are the Jones matrices of QWPs with slow axes along the 135° and 45° directions, respectively, and M_{mirror} is the reflection matrix. Therefore, after passing through the apparatus, the Jones vector becomes

$$\vec{E}_{1,rot} = \begin{bmatrix} 0 & i \\ -i & 0 \end{bmatrix} \cdot \begin{bmatrix} -\cos\theta \cdot e^{i\,p_1(x,y)} \\ \sin\theta \end{bmatrix}$$

$$= \begin{bmatrix} i \cdot \sin\theta \\ i \cdot \cos\theta \cdot e^{i\,p_1(x,y)} \end{bmatrix} \tag{S11}$$

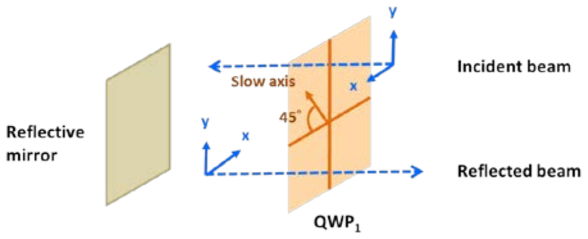

Figure S2. Illustration of the polarization rotation operation. QWP_1 here is identical to QWP_1 of Fig. 1 in the main text. Because of mirror reflection, the incident beam passes the QWP_1 with its slow axis oriented at 45° and the reflected beam oriented at 135° relative to the x axis.

After polarization rotation, the beam is aligned such that it is incident onto the area 2 of the SLM with the help of the reflective 4f system. During the second modulation, the x component is encoded by a phase factor of $e^{i\,p_2(x,y)}$, leaving the y component unaffected. Thus, the Jones vector of the reflected beam is evaluated as follows

$$\vec{E}_2 = \begin{bmatrix} E_{2,x} \\ E_{2,y} \end{bmatrix} = \begin{bmatrix} -1 & 0 \\ 0 & 1 \end{bmatrix} \cdot \begin{bmatrix} i \cdot \sin\theta \cdot e^{i\,p_2(x,y)} \\ i \cdot \cos\theta \cdot e^{i\,p_1(x,y)} \end{bmatrix}$$

$$= i \cdot \begin{bmatrix} -\sin\theta \cdot e^{i\, p_2(x,y)} \\ \cos\theta \cdot e^{i\, p_1(x,y)} \end{bmatrix}$$

$$= i \cdot (-\sin\theta \cdot e^{i\, p_2(x,y)}\, \hat{e}_x + \cos\theta \cdot e^{i\, p_1(x,y)}\, \hat{e}_y) \qquad \text{(S12)}$$

where the subscript "2" denotes the second modulation and two linear polarization eigenstates are denoted by $\hat{e}_x$ and $\hat{e}_y$, respectively. Hence, the last expression in equation (S12) represents the superposition of two linear polarization eigenstates. To transform these two linear eigenstates into another pair of eigenstates, the QWP_2 in the main text of Fig. 2 is used. We expand on this topic by dividing our discussion into three segments below.

(a) Linear polarization eigenstates

In the case of linearly polarized eigenstates, the slow axis of the QWP_2 is set to 0° with respect to the x axis. After passing through QWP_2, the complex amplitude of the emergent beam becomes

$$\vec{E} = i \cdot (-\sin\theta \cdot e^{-i\,\pi/4} \cdot e^{i\, p_2(x,y)} \cdot \hat{e}_x + \cos\theta \cdot e^{i\,\pi/4} \cdot e^{i\, p_1(x,y)} \cdot \hat{e}_y) \qquad \text{(S13)}$$

Let us now consider that both of the phase holograms encoded on the SLM have the following form of a helical phase distribution

$$p_i(\varphi) = m_i \cdot \varphi + \varphi_{i,0},\ i = 1, 2 \qquad \text{(S14)}$$

where m_i is the topological charge of the ith modulation, $\varphi_{i,o}$ is their corresponding phase offset, and φ is the azimuthal angle with respect to the x axis. Thus, after substituting equation (S14) into equation (S13) and, for simplicity, choosing their relative phase offset ($\varphi_{1,0}$ - $\varphi_{2,0}$) as -270°, some unimportant complex constants are canceled. Then, we can obtain the expression of VVBs spanned by two orthogonal linear polarization eigenstates

$$\begin{aligned}\vec{E} &= \cos\theta \cdot e^{i\, m_1 \varphi}\, \hat{e}_y + \sin\theta \cdot e^{i\, m_2 \varphi}\, \hat{e}_x \\ &= e^{i\,(m_1+m_2)\varphi/2} \left(\cos\theta \cdot e^{i\,(m_1-m_2)\varphi/2}\, \hat{e}_y + \sin\theta \cdot e^{-i\,(m_1-m_2)\varphi/2}\, \hat{e}_x\right)\end{aligned} \qquad \text{(S15)}$$

where $\hat{e}_x$ and $\hat{e}_y$ denote the linearly x- and y-polarized eigenstates with weights of $\sin\theta$ and $\cos\theta$, respectively. The angle of θ is concerned with the initial linearly polarized beam (see equation (S8)). To realize the SOP, if we substitute equation (S15) into equations (S3)-(S5), then we can obtain

$$S_1 = (\sin\theta)^2 - (\cos\theta)^2 \qquad \text{(S16)}$$

$$S_2 = 2\sin\theta \cdot \cos\theta \cdot \cos\big((m_1 - m_2)\varphi\big) \qquad \text{(S17)}$$

$$S_3 = 2\sin\theta \cdot \cos\theta \cdot \sin\big((m_1 - m_2)\varphi\big) \qquad \text{(S18)}$$

These results imply that all of the Stokes parameters of SOP on the transverse plane can be completely described by a geodesic path with radius of $2|\sin\theta||\cos\theta|$, which is located on the plane of $S_1 = (\sin\theta)^2 - (\cos\theta)^2$ intersected with the Poincaré sphere, as illustrated in Fig. S3. The blue (red) ellipses represent right- (left-) handed polarization states, and angle φ denotes the azimuthal coordinate of the beam and the plane formed by S_2 and S_3 axes on the sphere. Both of orientation (ψ) and ellipticity (χ) angles of

polarization ellipses can be obtained by substituting equations (S2)-(S5) into equations (S6) and (S7). The simulated results of ψ and χ are shown in Fig. S3. In the figure, one can control SOP not only by adjusting the value of θ leading to shifting the geodesic path on the sphere but also the value of $(m_1 - m_2)$. The smaller the θ value, the nearer to the y-polarized eigenstate the geodesic path becomes. That is, all of the orientation angles of elliptical polarizations are close to 90°. For each value θ, the larger the value of $|m_1 - m_2|$, the larger the azimuthal gradient of SOP on the transverse plane become; in addition, the handedness depends on the sign of $(m_1 - m_2)$. All of these results show that SOP of the beam have cylindrical symmetry because there is only one variable φ in equation (S13).

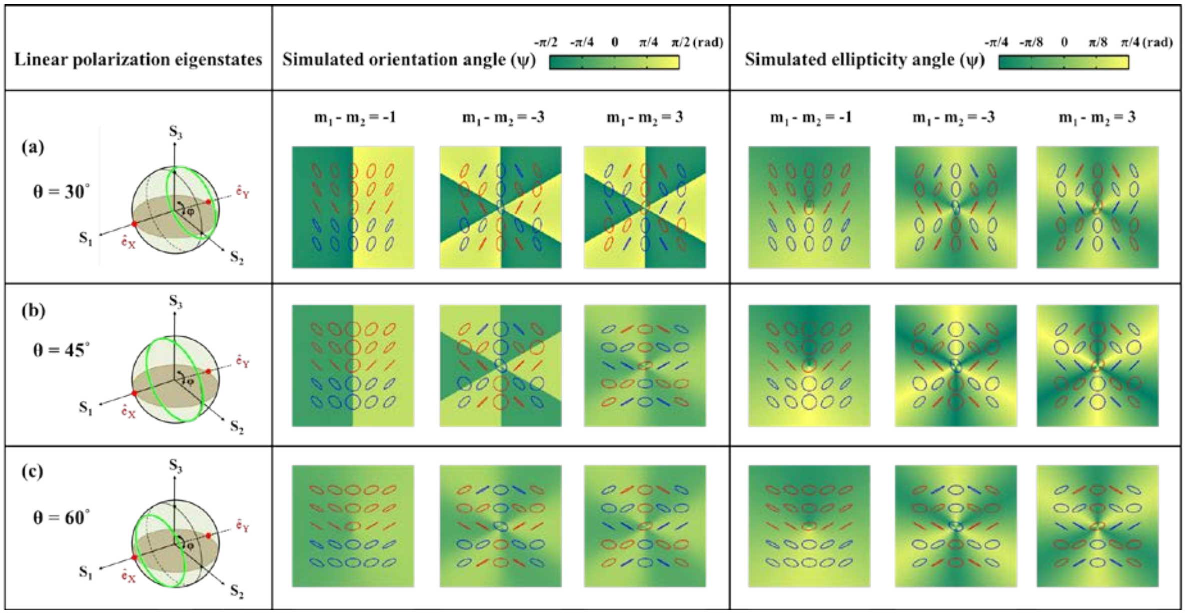

Figure S3. Illustration of the use of a Poincaré sphere to map SOP on the transverse plane. The geodesic path marked on the sphere corresponds to how SOP vary with the coordinate φ, which is denoted as the azimuthal coordinate on the transverse plane, as well as the plane formed by the S_2 and S_3 axes. Two linear polarization eigenstates are denoted by $\hat{e}_X$ and $\hat{e}_Y$, respectively, and the blue and red ellipses represent right- and left-handed polarizations, respectively.

(b) Circular polarization eigenstates

To convert linearly polarized eigenstates into circularly polarized eigenstates, the slow axis of QWP2 should be set to 45° with respect to the x axis. After considering the case of helical phase modulation and choosing $(\varphi_{1,0} - \varphi_{2,0})$ as -90°, the VVBs spanned by two orthogonal circular polarization eigenstates have the following form

$$\begin{aligned}\vec{E} &= \cos\theta \cdot e^{i m_1 \varphi}\, \hat{e}_R + \sin\theta \cdot e^{i m_2 \varphi}\, \hat{e}_L \\ &= e^{i(m_1+m_2)\varphi/2}\left(\cos\theta \cdot e^{i(m_1-m_2)\varphi/2}\, \hat{e}_R + \sin\theta \cdot e^{-i(m_1+m_2)\varphi/2}\, \hat{e}_L\right)\end{aligned} \tag{S19}$$

where $\hat{e}_R = \frac{1}{\sqrt{2}}\begin{bmatrix}1\\ i\end{bmatrix}$ and $\hat{e}_L = \frac{1}{\sqrt{2}}\begin{bmatrix}1\\ -i\end{bmatrix}$ denote the RCP and LCP eigenstates with weights of $\cos\theta$ and $\sin\theta$, respectively. Simulated results based on equation (S19) are shown in Fig. S4. The blue (red) ellipses represent right- (left-) handed polarization states, the black marks represent the linear polarizations on the transverse plane, and angle φ denotes the azimuthal coordinate of the beam as well as the plane formed by S_1 and S_2 axes on the sphere. Similar to the previous results, the angle θ controls the weights of each eigenstate, and therefore it determines both of the ellipticity and the helicity of polarization ellipses. The relationship

between m_1 and m_2 determines not only the spatial orientation distributions of ellipses but also the polarization mode. For example, the condition (m_1 - m_2) > 0 corresponds to the polarization anti-vortex mode [S2], also called π-vector beams [S3]. In particular, when θ = 45°, a special case in which only linear polarizations appear may be observed because of the equal weighted superposition of two eigenstates. In this special case, we can obtain CVBs by adjusting the phase offset ($\varphi_{1,0}$ - $\varphi_{2,0}$).

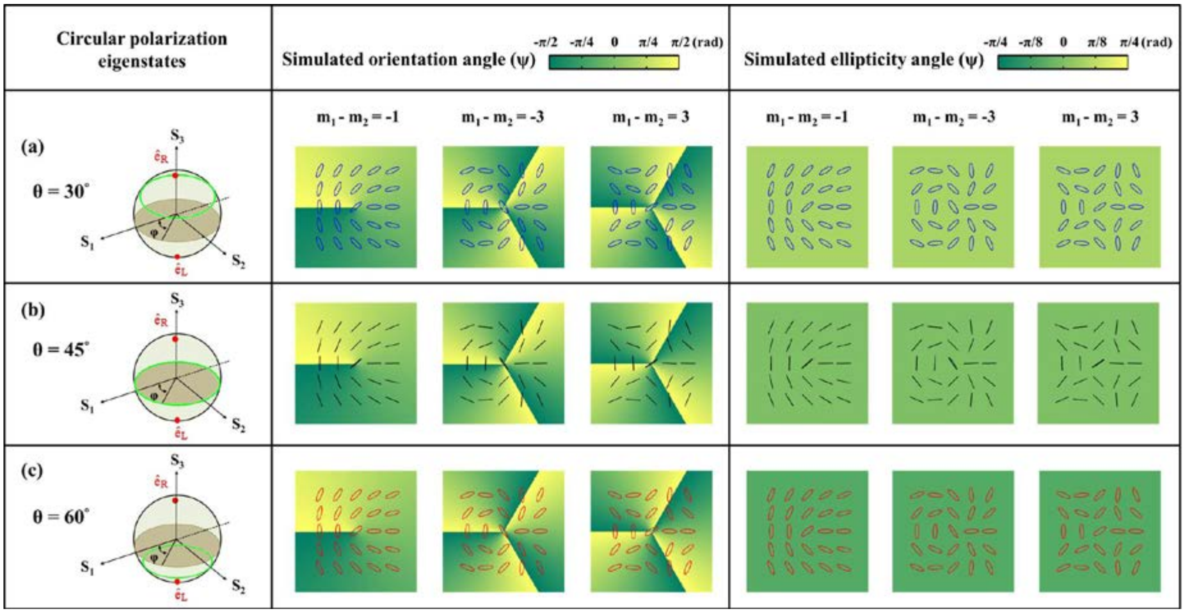

Figure S4. Illustration of the use of a Poincaré sphere to map SOP on the transverse plane. The geodesic path marked on the sphere corresponds to how SOP vary with the coordinate φ, which is denoted as the azimuthal coordinate on the transverse plane, as well as the plane formed by the S_1 and S_2 axes. Two circular polarization eigenstates of LCP and RCP are denoted by $\hat{e}_L$ and $\hat{e}_R$, respectively, and the blue and red ellipses represent right- and left-handed polarizations, respectively.

(c) Elliptical polarization eigenstates

In this case, the slow axis of QWP_2 could be set to any arbitrary angle other than 0° and 45°. After considering the case of helical phase modulation and choosing ($\varphi_{1,0}$ - $\varphi_{2,0}$) as -180°, VVBs spanned by two orthogonal elliptical polarization eigenstates have the following form

$$\begin{aligned}\vec{E} &= \cos\theta \cdot e^{i m_1 \varphi}\, \hat{e}_1 + \sin\theta \cdot e^{i m_2 \varphi}\, \hat{e}_2 \\ &= e^{i(m_1+m_2)\varphi/2}\left(\cos\theta \cdot e^{i(m_1-m_2)\varphi/2}\, \hat{e}_1 + \sin\theta \cdot e^{-i(m_1+m_2)\varphi/2}\, \hat{e}_2\right)\end{aligned} \tag{S20}$$

where $\hat{e}_1$ and $\hat{e}_2$ represent two orthogonal elliptical eigenstates with coefficients of $\sin\theta$ and $\cos\theta$, respectively. As an example, if we take the slow axis of QWP_2 to be 22.5°, the corresponding elliptical eigenstates are given in Fig. S5. Simulated results of SOP according to equation (S20) are shown in Fig. S6. The blue (red) ellipses represent right- (left-) handed polarization states, and angle φ denotes the azimuthal coordinate of the beam as well as the u-v plane, which intersects with the Poincaré sphere. As can be seen in the figure, only within a limited range of θ will the geodesic path simultaneously pass through the northern and southern hemispheres, resulting in alternation of right- and left-handed polarizations on the transverse plane. The alternation times depend on the absolute value of ($m_1 - m_2$) whereas the handedness on it sign.

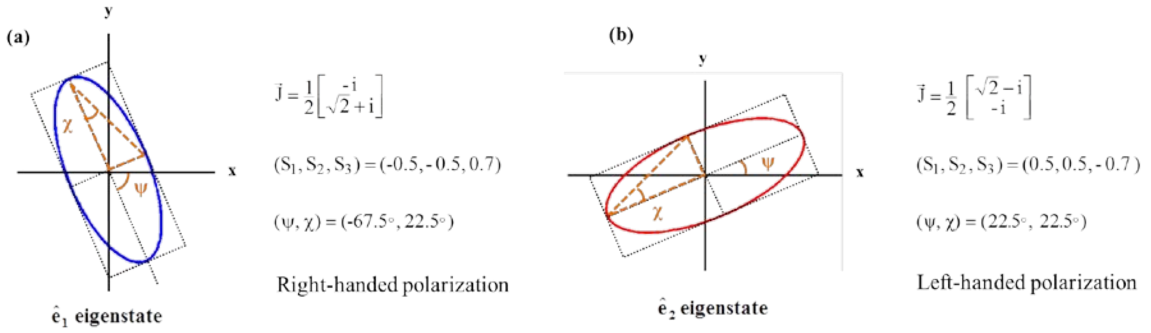

Figure S5. Illustration of two elliptical eigenstates in the case of the slow axis of QWP_2 oriented at 22.5°. The blue and red ellipses represent right- and left-handed polarizations, respectively. Detailed description of polarization parameters such as Jones vector, Stokes parameters, and orientation and ellipticity angles are also listed beside.

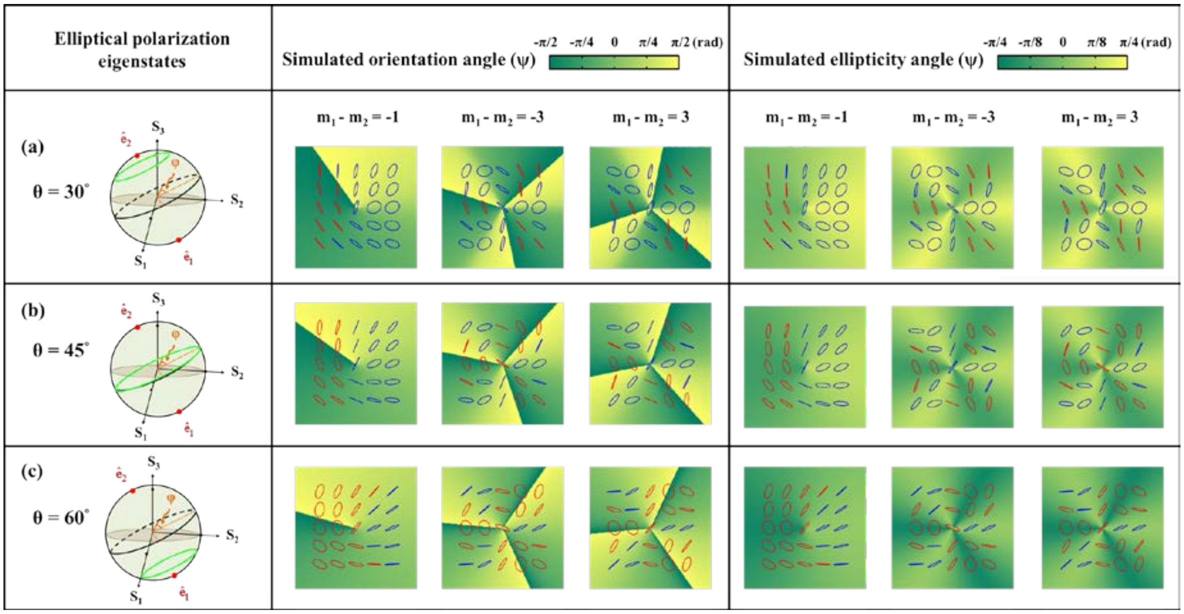

Figure S6. Illustration of the use of a Poincaré sphere to map SOP on the transverse plane. The geodesic path marked on the sphere corresponds to how SOP vary with the coordinate φ, which is denoted as the azimuthal coordinate on the transverse plane as well as the plane formed by the u and v axes. Two elliptical eigenstates are denoted by $\hat{e}_1$ and $\hat{e}_2$, respectively, and the blue and red ellipses represent right- and left-handed polarizations, respectively.

C. Measurement of the extinction ratio of a spatial light modulator

The Jones vector of an output beam $\vec{E}_{out}$ reflected off a parallel aligned liquid crystal SLM between two crossed polarizers can be obtained using the Jones matrix calculation

$$\vec{E}_{out} = P_2 \cdot W_{SLM} \cdot \vec{E}_{in} \quad \text{(S21)}$$

$$\vec{E}_{in} = \begin{bmatrix} \cos\theta \\ \sin\theta \end{bmatrix} \quad \text{(S22)}$$

$$W_{SLM} = \begin{bmatrix} e^{i\phi} & 0 \\ 0 & 1 \end{bmatrix} \quad \text{(S23)}$$

$$P_2 = \begin{bmatrix} \cos^2(\theta+\pi/2) & \sin(\theta+\pi/2)\cdot\cos(\theta+\pi/2) \\ \sin(\theta+\pi/2)\cdot\cos(\theta+\pi/2) & \sin^2(\theta+\pi/2) \end{bmatrix} \quad \text{(S24)}$$

where $\vec{E}_{in}$ is the Jones vector of an unpolarized incident beam after passing through the front polarizer (P_1), which is orientated at θ with respect to the x-axis; W_{SLM} is the Jones matrix of the SLM with liquid crystal molecules aligned in the x direction; the phase retardation of the SLM is $\phi = 2\pi d\Delta n/\lambda$, where Δn is the birefringence of the liquid crystal, d is the cell thickness, and λ is the wavelength of the incident beam; P_2 is the Jones matrix of the second polarizer (P_2) orientated at $(90^\circ + \theta)$ with respect to the x-axis. Moreover, it can be shown that the intensity of the output beam (I_{out}) is

$$I_{out} = \left|\vec{E}_{out}\right|^2 = \sin^2(2\theta)\sin^2(\frac{\phi}{2}) \quad \text{(S25)}$$

Accordingly, the maximum intensity occurs when $\theta = \pi/4$ and $\phi = \pi$, whereas the minimum occurs when $\theta = \pi/2$ and $\phi = \pi$, i.e., the SLM now acts as an HWP. It follows that the extinction ratio ΔP of the SLM can be defined as $\Delta P \equiv 10\cdot\log(I_{min}/I_{max})$ [S4]. Figure S7(a) shows the experimental setup for the extinction ratio measurement. To ensure that the SLM acts as a HWP, we have applied several voltages (gray levels) to the SLM to confirm that the phase retardation $\phi = \pi$. Figure S7(b) shows the measured results of intensity I_{out} for different values of θ, which is the orientation angle of the front polarizer (P_1), when $\phi = \pi$. In this figure, the maximum intensity (1) and the minimum intensity (0.0156) occur when $\theta = 45°$ and 90°, respectively. Thus, the extinction ratio of our SLM is approximately −18dB. This implies that the y-component will also be phase modulated in each phase modulation path, and it may cause errors in the polarization control of VVBs.

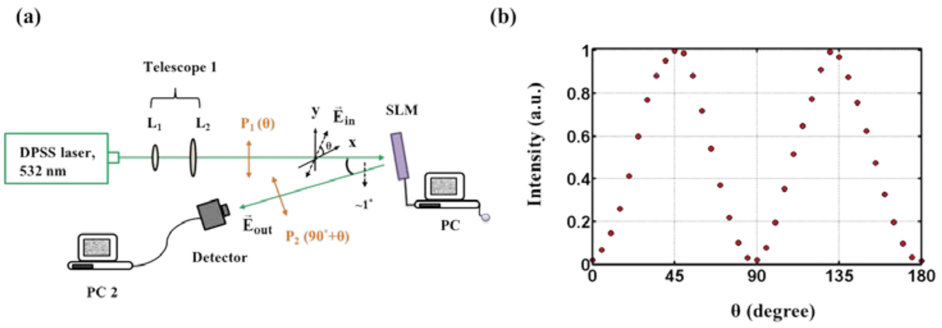

Figure S7. Experiment for measuring the extinction ratio of a SLM: (a) experimental setup. P_1, P_2: polarizer, HWP: half-wave plate; (b) measured intensity of output beam with different orientation angles θ.

D. Theoretical predication of orbital angular momentum of vectorial vortex beams

A convenient representation of vectorial vortex beams is achieved in the Lorenz gauge using the vector potential $\vec{A}(x, y, t)$ [S5]

$$\vec{A}(x,y,t)=\frac{i}{\omega}\left(\alpha(x,y)\hat{e}_x+\beta(x,y)\hat{e}_y\right)e^{i(\omega t-kz)} \tag{S26}$$

It can be shown that the complex amplitudes of the electric and magnetic field components of VVBs under the paraxial limit are

$$\begin{aligned}\vec{E}(x,y)&=-\frac{ic^2}{\omega}\nabla\times\vec{B}(x,y)\\&=\alpha(x,y)\hat{e}_x+\beta(x,y)\hat{e}_y-\frac{i}{k}\left(\frac{\partial}{\partial x}\alpha(x,y)+\frac{\partial}{\partial y}\beta(x,y)\right)\hat{e}_z\end{aligned} \tag{S27}$$

$$\begin{aligned}\vec{B}(x,y)&=\nabla\times\vec{A}(x,y)\\&=-\frac{k}{\omega}\left(\beta(x,y)\hat{e}_x-\alpha(x,y)\hat{e}_y+\frac{i}{k}\left(\frac{\partial}{\partial y}\alpha(x,y)-\frac{\partial}{\partial x}\beta(x,y)\right)\hat{e}_z\right)\end{aligned} \tag{S28}$$

The linear momentum density $\vec{p}$, which is defined as $\vec{p}=\varepsilon_o\vec{E}\times\vec{B}$, can be expressed and divided into transverse and longitudinal components

$$\vec{p}_\perp(x,y)=\frac{i\varepsilon_o}{4\omega}\left(\alpha^*\nabla\alpha+\beta^*\nabla\beta-\alpha\nabla\alpha^*-\beta\nabla\beta^*+\nabla\times[(\alpha^*\beta-\alpha\beta^*)\hat{z}]\right) \tag{S29}$$

$$p_z(x,y)=\frac{k\varepsilon_o}{2\omega}\left(|\alpha|^2+|\beta|^2\right) \tag{S30}$$

In addition, the φ-component of the transverse component $\vec{p}_\perp$ is

$$p_\varphi(x,y)=\frac{i\varepsilon_o}{4\omega}\frac{1}{r}\left(\alpha^*\frac{\partial}{\partial\varphi}\alpha+\beta^*\frac{\partial}{\partial\varphi}\beta-\alpha\frac{\partial}{\partial\varphi}\alpha^*-\beta\frac{\partial}{\partial\varphi}\beta^*-r\left(\frac{\partial}{\partial r}(\alpha^*\beta-\alpha\beta^*)\right)\right) \tag{S31}$$

Furthermore, the energy density of the beam is

$$u=c\cdot p_z=\frac{ck\varepsilon_o}{2\omega}\left(|\alpha|^2+|\beta|^2\right) \tag{S32}$$

The product of p_φ with r (radial distance with respect to the beam center) gives the z component of orbital angular momentum (OAM) density. In particular, j_z can be divided into spin and orbital parts

$$j_z(x,y)=r\cdot p_\varphi=j_z^{spin}+j_z^{orbital} \tag{S33}$$

$$j_z^{spin}=\frac{-i\varepsilon_o}{4\omega}r\frac{\partial}{\partial r}\left(\alpha^*\beta-\alpha\beta^*\right) \tag{S34}$$

$$j_z^{orbital}=\frac{i\varepsilon_o}{4\omega}\left(\alpha^*\frac{\partial}{\partial\varphi}\alpha+\beta^*\frac{\partial}{\partial\varphi}\beta-\alpha\frac{\partial}{\partial\varphi}\alpha^*-\beta\frac{\partial}{\partial\varphi}\beta^*\right) \tag{S35}$$

The OAM charge of beams can be obtained by examining the local ratio of OAM density j_z to energy density u, i.e., $j_z^{orbital}/u$. As an example, we first consider the case in which VVBs are spanned by two orthogonal linear polarization eigenstates. A comparison of (S27) and (S15) reveals that

$$\alpha = \sin\theta\, e^{i \cdot m_2 \varphi} \tag{S36}$$

$$\beta = \cos\theta\, e^{i \cdot m_1 \varphi} \tag{S37}$$

After substituting (S36) and (S37) into (S32) and (S35), the values of $j_z^{orbital}$ and u are

$$j_z^{orbital} = \frac{\varepsilon_o}{2\omega}\left(m_1 \cos^2\theta + m_2 \sin^2\theta\right) \tag{S38}$$

$$u = \frac{\varepsilon_o}{2}\left(\cos^2\theta + \sin^2\theta\right) \tag{S39}$$

Thus, the value of $j_z^{orbital}/u$ is

$$\frac{j_z^{orbital}}{u} = \frac{m_1 \cos^2\theta + m_2 \sin^2\theta}{\omega} \equiv \frac{\ell}{\omega} \tag{S40}$$

$$\ell \equiv m_1 \cos^2\theta + m_2 \sin^2\theta \tag{S41}$$

where we define the local OAM charge ℓ by (S41). Moreover, the average OAM charge, which is defined as the ratio of OAM flux to energy flux over the transverse plane per unit length of the beam, is

$$\langle \ell \rangle = \omega \frac{\iint j_z^{orbital}\, r\, dr\, d\phi}{\iint u\, r\, dr\, d\phi} = \ell \tag{S42}$$

Thus, (S42) implies that the average OAM charge is identical to the local one.

To date, we have discussed the OAM charge ℓ of VVBs spanned by two linear polarization eigenstates. In the following, we will further prove that the average OAM charge in (S41) is independent of the selection of polarization eigenstates. As aforementioned, to transform two linear polarization eigenstates into another pair of polarization eigenstates, the QWP_2 in the main text of Fig. 2 is used. In general, the Jones matrix of the QWP_2 can be written as

$$W_{QWP2} = \begin{bmatrix} w_1 & w_2 \\ -w_2^* & w_1^* \end{bmatrix} \tag{S43}$$

where $w_1 = e^{-i\Gamma/2}\cos^2\psi + e^{i\Gamma/2}\sin^2\psi$, $w_2 = -i\sin(\Gamma/2)\sin(2\psi)$, Γ is the phase retardation of the QWP_2, and ψ is the orientation angle of the slow axis of QWP. The expression of VVBs spanned by another pair of orthogonal polarization OAM eigenstates, denoted as $\vec{E}'$, can be obtained from the following

$$\vec{E}' = W_{QWP2} \cdot \left(\sin\theta\, e^{i m_2 \varphi}\hat{e}_x + \cos e^{i m_1 \varphi}\,\hat{e}_y\right)$$
$$= \begin{bmatrix} w_1 & w_2 \\ -w_2^* & w_1^* \end{bmatrix}\begin{bmatrix} \alpha \\ \beta \end{bmatrix} \quad \text{(S44)}$$
$$\equiv \begin{bmatrix} \alpha' \\ \beta' \end{bmatrix}$$

where α and β are given in (S36) and (S37), respectively, $\alpha' \equiv w_1\alpha + w_2\beta$ and $\beta' \equiv -w_2^*\alpha + w_1^*\beta$. The normalization condition $\left|\alpha'\right|^2 + \left|\beta'\right|^2 = 1$ still holds because the Jones matrix of a wave plate is a unitary matrix. After substituting (S44) into (S32) and (S35), we obtain that

$$u' = \frac{ck\varepsilon_o}{2\omega}\left(\left|\alpha'\right|^2 + \left|\beta'\right|^2\right) \quad \text{(S45)}$$

$$j_z'^{\,orbital} = \frac{i\varepsilon_o}{4\omega}\left(\alpha'^*\frac{\partial}{\partial\varphi}\alpha' + \beta'^*\frac{\partial}{\partial\varphi}\beta' - \alpha'\frac{\partial}{\partial\varphi}\alpha'^* - \beta'\frac{\partial}{\partial\varphi}\beta'^*\right)$$
$$= \frac{i\varepsilon_o}{4\omega}\left(\alpha^*\frac{\partial}{\partial\varphi}\alpha + \beta^*\frac{\partial}{\partial\varphi}\beta - \alpha\frac{\partial}{\partial\varphi}\alpha^* - \beta\frac{\partial}{\partial\varphi}\beta^*\right) \quad \text{(S46)}$$

The above results are the same as (S32) and (S35), which are derived under the linear polarization eigenstates. As a result, we conclude that the OAM charge of VVBs is invariant under the linear transformation of polarization eigenstates using the QWP_2.

References

[S1] Saleh, B. E. A. & Teich, M. C. *Fundamentals of Photonics*. (Wiley, 1991).

[S2] Christian, M., Alexander, J., Severin, F., Stefan, B. & Monika, R.-M. Tailoring of arbitrary optical vector beams. *New J. Phys.* **9**, 78 (2007).

[S3] Milione, G., Sztul, H. I., Nolan, D. A. & Alfano, R. R. Higher-Order Poincaré Sphere, Stokes Parameters, and the Angular Momentum of Light. *Phys. Rev. Lett.* **107**, 053601 (2011).

[S4] X. Hu, O. Hadaler, and H. J. Coles, "High Optical Contrast Liquid Crystal Switch and Analogue Response Attenuator at 1550 nm," *IEEE Photonics Technology Letters* **23**, 1655-1657 (2011).

[S5] D. Zhang, X. Feng, K. Cui, F. Liu, and Y. Huang, "Identifying Orbital Angular Momentum of Vectorial Vortices with Pancharatnam Phase and Stokes Parameters," *Sci. Rep.* **5**, 11982 (2015).
